# Mr. Henry Edgar Valentine Blake

**Published:** 1910-06-11

**Authors:** 


The death is reported, in Dublin, of Mr. Henry Edgar Valentine Blake, of Renvyle. Mr. Blake, who had studied in Ireland, became a licentiate of the Irish Royal Colleges of Medicine and Surgery, and an L.M. in 1897.

